# Factors associated with older adults’ knowledge, attitude and practice on skin cancer prevention

**DOI:** 10.1590/0034-7167-2022-0606

**Published:** 2023-10-09

**Authors:** Antonia Imaculada Santos Serafim, Samia Jardelle Costa de Freitas Maniva, Regina Kelly Guimarães Gomes Campos, Paulo Goberlânio de Barros Silva, Patrícia Neyva da Costa Pinheiro, Consuelo Helena Aires de Freitas Lopes, Huana Carolina Cândido Morais, Aline de Oliveira de Freitas

**Affiliations:** I Centro Universitário INTA. Sobral, Ceará, Brazil; II Universidade Federal do Ceará. Fortaleza, Ceará, Brazil; III Universidade Estadual do Ceará. Fortaleza, Ceará, Brazil; IV Universidade da Integração Internacional da Lusofonia Afro-Brazileira. Acarape, Ceará, Brazil

**Keywords:** Skin Neoplasms, Aged, Knowledge, Attitude, Nursing, Neoplasias Cutáneas, Anciano, Conocimiento, Actitud, Enfermería, Neoplasias Cutâneas, Idoso, Conhecimento, Atitude, Enfermagem.

## Abstract

**Objectives:**

to identify factors associated with older adults’ knowledge, attitude and practice regarding skin cancer prevention.

**Methods:**

this is a cross-sectional study, carried out with 120 older adults from a Basic Health Unit in Quixadá, Ceará, from September to November 2018.

**Results:**

individuals aged 60 to 69 years and working were statistically associated with adequate knowledge (p=0.038). Having light skin, eyes and hair was associated with adequate attitude (p=0.030). Having skin problems, such as bleeding wounds, was associated with adequate practice (p=0.016). With regard to inappropriate behavior for skin cancer prevention, there was a statistically significant association between working or having worked under direct exposure to the sun, inadequate knowledge (p=0.036), inadequate attitude (p=0.010) and having incomplete primary education and inadequate practice (p<0.001).

**Conclusions:**

sociodemographic and clinical factors influence older adults’ knowledge, attitude and practice regarding skin cancer prevention.

## RESUMO

**Objetivos:**

identificar fatores associados a conhecimento, atitude e prática de idosos sobre a prevenção do câncer de pele.

**Métodos:**

estudo transversal, realizado com 120 idosos de uma Unidade Básica de Saúde em Quixadá, Ceará, de setembro a novembro de 2018.

**Resultados:**

indivíduos na faixa etária de 60 a 69 anos e trabalhando estiveram associados estatisticamente ao conhecimento adequado (p=0,038). O fato de ter pele, olhos e cabelos claros estava associado à atitude adequada (p=0,030). Ter problemas de pele, do tipo ferida sangrante, estava associado à prática adequada (p=0,016). Acerca das condutas inadequadas para a prevenção do câncer de pele, observou-se associação estatisticamente significante entre trabalhar ou já ter trabalhado sob exposição direta ao sol, conhecimento inadequado (p=0,036), atitude inadequada (p=0,010), ter o ensino fundamental incompleto e prática inadequada (p<0,001).

**Conclusões:**

fatores sociodemográficos e clínicos influenciam no conhecimento, atitude e prática de idosos sobre a prevenção do câncer de pele.

## RESUMEN

**Objetivos:**

identificar factores asociados al conocimiento, actitud y práctica de los ancianos en relación a la prevención del cáncer de piel.

**Métodos:**

estudio transversal, realizado con 120 ancianos de una Unidad Básica de Salud de Quixadá, Ceará, de septiembre a noviembre de 2018.

**Resultados:**

los individuos de 60 a 69 años y que trabajaban se asociaron estadísticamente con conocimientos adecuados (p=0,038). Tener piel, ojos y cabello claros se asoció con una actitud adecuada (p=0,030). Tener problemas en la piel, como heridas sangrantes, se asoció con una práctica adecuada (p=0,016). Sobre conductas inapropiadas para la prevención del cáncer de piel, hubo asociación estadísticamente significativa entre trabajar o haber trabajado bajo exposición directa al sol, conocimientos inadecuados (p=0,036), actitud inadecuada (p=0,010), tener instrucción primaria incompleta y práctica inadecuada (p<0,001).

**Conclusiones:**

los factores sociodemográficos y clínicos influyen en el conocimiento, la actitud y la práctica de los ancianos respecto a la prevención del cáncer de piel.

## INTRODUCTION

The term “cancer” is comprehensive and corresponds to more than 100 different types of malignant diseases that have as a common characteristic the disorderly growth of cells, which can invade adjacent tissues or organs at a distance^(1)^.

It is a public health problem, especially in developing countries. Estimates show that in the year 2020, there were 309,750 cases of cancer in men and 316,280 cases in women. Among the types of cancer, skin cancer stands out. One out of every three cancers diagnosed is skin cancer^(2)^. In Brazil, non-melanoma skin cancer is the most frequent, with high cure rates when detected and treated early^(3)^. There is a higher prevalence in older adults^(4)^.

Melanoma skin cancer is less frequent and has a worse prognosis, with high mortality. When detection is early, the chances of cure reach more than 90%^(2,5)^. It resembles moles, eczema or other benign lesions. For early detection, skin self-examination and detailed assessment by health professionals are recommended^(6)^. For this type of cancer, population aging and ultraviolet exposure are contributing factors to the increase in its incidence^(7)^.

Currently, the world population is undergoing a demographic transition, reflecting the growth of older adults. In Brazil, older adults are defined as people aged 60 or over. This is the sixth country with the highest number of older adults. In the last 60 years, there has been an increase of 15 million older adults in the country, from 4% to 9%, representing an increase of 33 million^(8-9)^.

Old age increases the chances of people developing skin cancer^(7)^. Another risk factor is chronic and excessive exposure to sunlight, which contains significant amounts of ultraviolet (UV) rays. These rays trigger different pathological changes in the skin. As a result, wearing sunscreen is recommended to prevent UV-induced skin damage and photoaging^(10-11)^.

Analyzing the factors related to older adults’ knowledge, attitude and practice (KAP) about skin cancer prevention can be an opportune measure to plan and assess actions aimed at health education. The KAP model is based on the assumption that health behavior is linked to a sequential process: it originates in the acquisition of scientifically correct knowledge, which leads to the formation of a favorable attitude and the adoption of a health practice^(12)^. Therefore, appropriate attitudes and practices can mitigate the risks of appearance of cancerous lesions, contributing to skin neoplasm prevention^(13)^.

Nursing plays a relevant role in health promotion and skin cancer prevention and treatment in care spaces. Therefore, it is necessary for nurses to be able to recognize and teach the population about the clinical manifestations of this type of neoplasm, in order to favor the early recognition and identification of suspected cases, in addition to setting goals to minimize the consequences of illness faced by people with cancer and their families, promoting quality of life and continuity of care^(14)^.

Given the above considerations, the present study is justified by skin cancer mortality and morbidity. There was a lack of knowledge about studies that address knowledge, attitudes and practices related to skin cancer prevention in older adults. It is expected that the study data can guide health professionals and managers about the importance of promoting educational campaigns for skin cancer prevention in older adults.

## OBJECTIVES

To identify factors associated with older adults’ KAP regarding skin cancer prevention.

## METHODS

### Ethical aspects

The study was conducted in accordance with national^(15)^ and international ethical guidelines, and was approved by the Research Ethics Committee of *Centro Universitário Católica de Quixadá*, whose opinion is attached to this submission.

### Study design, period and place

This is a cross-sectional study, structured on the KAP survey methodology in relation to skin cancer prevention^(14)^, carried out from September to December 2018, in a Basic Health Unit in the city of Quixadá, Ceará, Brazil. To direct the presentation of information, the STrengthening the Reporting of OBservational studies in Epidemiology (STROBE) were considered^(16)^.

### Population or sample; inclusion and exclusion criteria

Sampling was simple random for convenience. Older adults aged 60 years or older who spontaneously agreed to participate in the research were included. Older adults who had communication limitations that made it impossible to respond to the data collection form and those who interrupted the interview and did not answer all the questions on the adopted form were excluded. To obtain the sample calculation, the formula for a finite population was used, based on the 250 older adults registered in the unit selected for the study, and a confidence level of 95% was considered, an estimated prevalence of the investigated phenomena of 50% and a margin of error of 5%, resulting in a sample size of 152 participants. However, due to logistical issues related to the non-attendance of older adults to the health unit during the data collection period, a convenience sample of 120 participants was obtained.

### Study protocol

The research protocol used comprised the application of an instrument with sociodemographic and clinical information, prepared by the researchers, which supported the definition of variables: age; sex; education; profession; marital status; reported chronic illnesses; use and types of medications; having skin problem; types of skin problems reported; working or having worked under sun exposure; having light skin, eyes and hair. The age variable was processed as continuous, and the others as categorical.

The KAP survey form, containing questions that assessed older adults’ KAP about skin cancer prevention, was constructed based on studies^(17-19)^. After elaboration, the instrument was submitted to analysis by three evaluators: a dermatologist and two PhD nurse with experience in using the KAP survey. The listed suggestions were incorporated into the final version of the instrument. The KAP survey was selected for identifying data from a specific population that clarify knowledge, attitudes and biopsychosocial practices, showing ways for a more effective intervention^(20)^.

The variables on KAP were defined as follows: 1) adequate knowledge: when older adults mentioned having already heard about skin cancer, skin cancer prevention and knowing methods for skin cancer prevention; 2) adequate attitude: when older adults believe that wearing sunscreen, a dermatological consultation at least once a year, using protective clothing and skin self-examination are actions that are always necessary for skin cancer prevention; 3) adequate practice: when older adults report wearing sunscreen with a protection factor of at least 15, with reapplication of sunscreen every two hours, at least, in addition to using a wide-brimmed hat, glasses, long-sleeved pants and T-shirt, regular observation own skin, and avoid sun exposure between 10 and 16 hours^(7,18,21)^. Inappropriate conduct was considered when any of the items above were not mentioned or practiced by participants.

For data collection, an invitation was made to older adults to participate in the research, with the objectives of the study explained. The Informed Consent Form (ICF) was obtained from all individuals involved in the study in writing. Then, the form was applied, using the interview technique, which lasted an average of 15 minutes.

### Analysis of results, and statistics

Data were organized and tabulated in Microsoft Excel^®^ and exported to the Statistical Package for the Social Sciences (SPSS), version 20.0, for statistical analysis, adopting a significance level of 0.05. The absolute and percentage frequencies of each variable were calculated, and Fisher’s exact test was applied to verify the statistical association between variables.

## RESULTS

Sociodemographic data showed that the majority were female (57%), had incomplete elementary school (54%), were married (70%) and did not work (55%). The mean age was 69.8 years (± 4.7 years). As for skin cancer prevention, it was found that 68 (57%) had adequate knowledge, 52 (43%), inadequate knowledge, 70 (58%), adequate attitude, 50 (42%), inadequate attitude, 20 (17%), adequate practice, and 100 (83%), inadequate practice.

It was observed that the age range of 60 to 69 years and working was statistically associated with adequate knowledge (p=0.038). Among the older adults who worked, 37 (68%) declared themselves to be farmers. Having incomplete elementary school was associated with inadequate practice (p<0.001), and having completed high school (p<0.001) and completed higher education (p<0.001) was associated with adequate practice for skin cancer prevention. No significant association was identified between the frequency of KAP with sex and marital status (Table 1).

**Table 1:** Association of sociodemographic characteristics with older adults’ adequate knowledge, attitude and practice on skin cancer prevention, Quixadá, Ceará, Brasil, 2018

Sociodemographic variables		Adequate knowledge			Adequate attitude			Adequate practice		
	Total	No	Yes	*p* value	No	Yes	*p* value	No	Yes	*p* value
Age										
60 to 69	69 (57.5%)	21 (45.7%)	48 (64.9%)^*^	0.038	16 (48.5%)	53 (60.9%)	0.219	64 (57.1%)	5 (62.5%)	0.767
70+	51 (42.5%)	25 (54.3%)^*^	26 (35.1%)		17 (51.5%)	34 (39.1%)		48 (42.9%)	3 (37.5%)	
Sex										
Male	52 (43.3%)	16 (34.8%)	36 (48.6%)	0.136	12 (36.4%)	40 (46.0%)	0.343	50 (44.6%)	2 (25.0%)	0.279
Female	68 (56.7%)	30 (65.2%)	38 (51.4%)		21 (63.6%)	47 (54.0%)		62 (55.4%)	6 (75.0%)	
Education										
Illiterate	16 (13.3%)	9 (19.6%)	7 (9.5%)	0.149	7 (21.2%)	9 (10.3%)	0.116	16 (14.3%)	0 (0.0%)	**<0.001**
Elementary	19 (15.8%)	4 (8.7%)	15 (20.3%)		6 (18.2%)	13 (14.9%)		17 (15.2%)	2 (25.0%)	
Incomplete elementary school	65 (54.2%)	29 (63.0%)	36 (48.6%)		20 (60.6%)	45 (51.7%)		64 (57.1%)^*^	1 (12.5%)	
Complete high school	13 (10.8%)	3 (6.5%)	10 (13.5%)		0 (0.0%)	13 (14.9%)		11 (9.8%)	2 (25.0%)^*^	
Incomplete high school	3 (2.5%)	1 (2.2%)	2 (2.7%)		0 (0.0%)	3 (3.4%)		3 (2.7%)	0 (0.0%)	
Complete higher education	3 (2.5%)	0 (0.0%)	3 (4.1%)		0 (0.0%)	3 (3.4%)		1 (0.9%)	2 (25.0%)^*^	
Incomplete higher education	1 (0.8%)	0 (0.0%)	1 (1.4%)		0 (0.0%)	1 (1.1%)		0 (0.0%)	1 (12.5%)	
Occupation										
Working	54 (45.0%)	14 (30.4%)	40 (54.1%)^*^	0.011	12 (36.4%)	42 (48.3%)	0.242	53 (47.3%)	1 (12.5%)	0.056
Not working	66 (55.0%)	32 (69.6%)^*^	34 (45.9%)		21 (63.6%)	45 (51.7%)		59 (52.7%)	7 (87.5%)	
Marital status										
Married	84 (70.0%)	33 (71.7%)	51 (68.9%)	0.797	25 (75.8%)	59 (67.8%)	0.313	78 (69.6%)	6(75.0%)	0.452
Single	12 (10.0%)	3 (6.5%)	9 (12.2%)		2 (6.1%)	10 (11.5%)		10 (8.9%)	2 (25.0%)	
Stable union	4 (3.3%)	1 (2.2%)	3 (4.1%)		0 (0.0%)	4 (4.6%)		4 (3.6%)	0 (0.0%)	
Divorced	4 (3.3%)	2 (4.3%)	2 (2.7%)		0 (0.0%)	4 (4.6%)		4 (3.6%)	0 (0.0%)	
Widower	16 (13.3%)	7 (15.2%)	9 (12.2%)		6 (18.2%)	10 (11.5%)		16 (14.3%)	0 (0.0%)	

* *p<0.05, Fisher’s exact test (n, %).*

Chronic diseases self-reported by older adults and continuous use medications did not show a statistically significant association with adequate KAP regarding skin cancer prevention. It was observed that the main chronic disease was hypertension (55.8%) and that 3.3% of older adults reported a previous diagnosis of skin cancer (Table 2).

**Table 2:** Association between the occurrence of reported chronic diseases and use of continuous medication with older adults’ adequate knowledge, attitude and practice regarding skin cancer prevention, Quixadá, Ceará, Brazil, 2018

Variables		Adequate knowledge			Adequate attitude			Adequate practice		
	Total	No	Yes	*p* value	No	Yes	*p* value	No	Yes	*p* value
Self-reported chronic diseases										
No chronic disease	15 (12.5%)	5 (10.9%)	10 (13.5%)	0.257	5 (15.2%)	10 (11.5%)	0.421	14 (12.5%)	1 (12.5%)	0.907
Hypertension	54 (45.0%)	17 (37.0%)	37 (50.0%)		16 (48.5%)	38 (43.7%)		49 (43.8%)	5 (62.5%)	
Diabetes	20 (16.7%)	8 (17.4%)	12 (16.2%)		4 (12.1%)	16 (18.4%)		19 (17.0%)	1 (12.5%)	
Skin cancer	4 (3.3%)	3 (6.5%)	1 (1.4%)		2 (6.1%)	2 (2.3%)		4 (3.6%)	0 (0.0%)	
Family history of cancer	8 (6.7%)	2 (4.3%)	6 (8.1%)		0 (0.0%)	8 (9.2%)		7 (6.3%)	1 (12.5%)	
Arthritis/rheumatism	9 (7.5%)	5 (10.9%)	4 (5.4%)		4 (12.1%)	5 (5.7%)		9 (8.0%)	0 (0.0%)	
Chronic pain in the spine	8 (6.7%)	4 (8.7%)	4 (5.4%)		2 (6.1%)	6 (6.9%)		8 (7.1%)	0 (0.0%)	
Heart disease	2 (1.7%)	2 (4.3%)	0 (0.0%)		0 (0.0%)	2 (2.3%)		2 (1.8%)	0 (0.0%)	
Continuous use medication										
Yes	94 (78.3%)	35 (76.1%)	59 (79.7%)	0.430	27 (81.8%)	67 (77.0%)	0.740	88 (78.6%)	6 (75.0%)	0.925
No	25 (20.8%)	10 (21.7%)	15 (20.3%)		6 (18.2%)	19 (21.8%)		23 (20.5%)	2 (25.0%)	
Types of medications										
No	25 (20.8%)	10 (21.7%)	15 (20.3%)	0.305	6 (18.2%)	19 (21.8%)	0.898	23 (20.5%)	2 (25.0%)	0.693
Antihypertensive	59 (49.2%)	21 (45.7%)	38 (51.4%)		16 (48.5%)	43 (49.4%)		54 (48.2%)	5 (62.5%)	
Antidiabetic	25 (20.8%)	8 (17.4%)	17 (23.0%)		7 (21.2%)	18 (20.7%)		24 (21.4%)	1 (12.5%)	
Analgesics/anti-inflammatories	11 (9.2%)	7 (15.2%)	4 (5.4%)		4 (12.1%)	7 (8.0%)		11 (9.8%)	0 (0.0%)	

* *p<0.05, Fisher’s exact test (n, %).*

Working or having worked under direct exposure to the sun showed a statistically significant association with not having adequate knowledge (p=0.036) and not having an adequate attitude (p=0.010) for skin cancer prevention. Having light skin, eyes and hair was associated with having an adequate attitude (p=0.030). Having a skin problem, such as skin peeling and reddish patches with crusts, was associated with inadequate practice (p=0.016). Meanwhile, having a bleeding wound was significantly associated with having adequate practice (p=0.016) (Table 3).

**Table 3:** Association of clinical characteristics with older adults’ adequate knowledge, attitude and practice on skin cancer prevention, Quixadá, Ceará, Brazil, 2018

Variables		Adequate knowledge			Adequate attitude			Adequate practice		
	Total	No	Yes	*p* value	No	Yes	*p* value	No	Yes	*p* value
Self-reported skin problems										
Yes	25 (20.8%)	13 (28.3%)	12 (16.2%)	0.114	10 (30.3%)	15 (17.2%)	0.116	23 (20.5%)	2 (25.0%)	0.764
No	95 (79.2%)	33 (71.7%)	62 (83.8%)		23 (69.7%)	72 (82.8%)		89 (79.5%)	6 (75.0%)	
Which skin problems										
No	96 (80.0%)	33 (71.7%)	63 (85.1%)	0.289	23 (69.7%)	73 (83.9%)	0.205	90 (80.4%)	6 (75.0%)	0.016
Skin peeling	9 (7.5%)	4 (8.7%)	5 (6.8%)		4 (12.1%)	5 (5.7%)		9 (8.0%)^*^	0 (0.0%)	
Pigmented patches/plaques with crusts	11 (8.3%)	7 (13.0%)	4 (5.4%)		6 (15.2%)	5 (5.7%)		11 (8.9%)^*^	0 (0.0%)	
Bleeding wound	5 (4.2%)	3 (6.5%)	2 (2.7%)		1 (3.0%)	4 (4.6%)		3 (2.7%)	2 (25.0%)^*^	
Works or has worked under direct sun exposure										
Yes	89 (74.2%)	39 (84.8%)^*^	50 (67.6%)	0.036	30 (90.9%)^*^	59 (67.8%)	0.010	85 (75.9%)	4 (50.0%)	0.106
No	31 (25.8%)	7 (15.2%)	24 (32.4%)^*^		3 (9.1%)	28 (32.2%)^*^		27 (24.1%)	4 (50.0%)	
Has light skin, eyes, hair										
Yes	44 (36.7%)	16 (34.8%)	28 (37.8%)	0.736	7 (21.2%)	37 (42.5%)^*^	0.030	39 (34.8%)	5 (62.5%)	0.117
No	76 (63.3%)	30 (65.2%)	46 (62.2%)		26 (78.8%)^*^	50 (57.5%)		73 (65.2%)	3 (37.5%)	

* *p<0.05, Fisher’s exact test.*

## DISCUSSION

With population aging and the increase in neoplasms^(7)^, it is essential to identify factors that may contribute to skin cancer prevention. This study sought to know the factors associated with older adults’ KAP regarding the prevention of this type of cancer.

It was found that the age group from 60 to 69 years old was associated with adequate knowledge for skin cancer prevention, while in the oldest older adults, aged over 70 years, inadequate knowledge was identified. This can be explained by the fact that, as age advances, there is a progressive loss of health^(22)^, with a greater risk of cognitive impairment and incidence of chronic conditions^(23-24)^.

There was a similar distribution regarding sex (57% women and 43% men), and no significant association was identified between this variable and the other variables investigated. Data showing sex differences in access to health services are already known, which show that women seek health services more, while men seek assistance when there is pain or a serious health problem^(25)^. Preventive measures and early diagnosis of skin cancer are necessary for both sexes^(26)^.

Low education was identified. Most older adults had incomplete elementary school, which was associated with inadequate practice for skin cancer prevention, while having completed high school or higher education was associated with adequate practice. The education of older adults prevalent in Brazil is incomplete elementary school, especially among older women and residents of rural areas^(27)^. A study points out that the higher the education, the greater the access to health services, and the better the living conditions during aging, with a lower incidence of diseases^(28)^. Such social inequalities have an impact on older adults’ health and well-being and therefore must be combated^(29)^.

There was a significant association between working and having adequate knowledge, at the same time not working was associated with inadequate knowledge. The growth of the working older adult population was driven by the new public pension, which raised the minimum retirement age for workers^(30)^. Aging is commonly associated with a decline in work ability. However, the continuity of older adults in work activities is related not only to the improvement of financial conditions, but is considered good for health. Therefore, quality of work is a fundamental issue^(31-32)^.

It was found that 57% of older adults had adequate knowledge regarding skin cancer prevention, 58% adequate attitude and 17% adequate practice. These data are worrying and should be considered in health promotion and preventive actions among older adults. By the logic of KAP, health behaviors depend on acquiring correct knowledge that leads to a favorable attitude that, in turn, can lead to healthy practices. However, several factors can influence the disposition for certain health practices, such as beliefs and cultural factors, access to health services and costs^(12)^. Given the above, it was not possible to determine why study participants had good knowledge and adequate attitude towards skin cancer prevention, but antagonistically had inadequate practice.

No statistically significant association was identified between older adults’ KAP with the presence of chronic diseases and medication use, although hypertension was self-reported by 55.8% of participants. It is known that, with the increase in longevity, two thirds of older adults have two or more chronic diseases^(33)^. The impact of hypertension on individuals’ lives is pointed out by studies^(34-36)^.

The occurrence of 3.3% of skin cancer was verified, through self-reported information (yes or no), and it was not possible to identify the type of neoplasm, whether melanoma or non-melanoma. Global incidence estimates^(37)^ indicate that skin cancer is the most frequent of all types of cancer, being more common in men than in women, varying between regions of the world^(38)^. However, a Brazilian study found that self-reported skin cancer by older adults at the first diagnosis was 13.9% (95%CI:9.1-20.6) among men and 17.3% (95%CI:14. 2-20.8) among women^(39)^. It should be noted that, often, neoplastic lesions in the early stages can be ignored by individuals because they resemble spots or moles, causing the medical assessment of skin lesions to be neglected^(40)^.

With regard to self-reported skin problems, having skin peeling and pigmented patches/plaques with crusts was statistically associated with poor practice. This can be explained by the fact that such skin changes can be the result of prolonged sun exposure, with the cumulative effect of ultraviolet rays, and lack of adherence to photoprotection measures^(6,41-42)^.

Also noteworthy is solar or actinic keratosis, characterized by reddish patches or plaques with a rough texture and frequent crusts on the face and arms of people over 65 years of age^(40,43-44)^. These are lesions with the potential for malignant transformation to non-melanoma skin cancer^(43)^. The older adults reported skin changes that resembled the description of actinic keratosis: “pigmented spots/plaques with crusts”. Actions aimed at early diagnosis are of great importance. In this field, nurses must carry out educational interventions for the early recognition of suggestive lesions^(45-46)^.

Having a bleeding wound was associated with adequate practice. The occurrence of bleeding is common in several skin disorders, including tumor wounds. The infiltration of neoplastic cells makes the lesions friable, facilitating bleeding^(46)^. The fact of having a bleeding wound can affect individuals, making them constantly remember the injury and even reveal it to other people^(47-48)^. It can be inferred that this contributed to the adoption of appropriate practices for skin cancer prevention by older adults.

It was found that 74.3% of older adults work or had already worked under direct exposure to the sun, which can be explained by the occupation of farmer, pointed out by the majority of older adults who carried out work activities. This variable showed a significant association with not having adequate knowledge. A study showed that the number of hours of occupational sun exposure is related to skin cancer^(49)^.

Health education is a strategy to increase the population’s knowledge and promote changes in behavior related to skin cancer, especially among older adults. Such action is promising for nursing in the various care settings^(46)^, and should be based on guidelines such as avoiding exposure to the sun without protection, especially between 10 am and 3 pm, using sunscreen in exposed areas, clothing and accessories such as sunglasses, hat, cap, visors and umbrella or umbrellas, aiming at photoprotection. Another measure is frequent skin self-examination of the entire body, looking for skin changes^(40)^.

It was observed that having light skin, eyes and hair was significantly associated with having an adequate attitude towards skin cancer, while not having light skin and eyes was associated with an inadequate attitude. This finding can be explained by the fact that people with such characteristics have skin that is more vulnerable to sunlight action. A study^(50)^ showed that lighter eye and hair color was associated with an increased risk of melanoma, basal cell carcinoma and squamous cell carcinoma.

### Study limitations

Despite the significant results, the study presented as a limitation the absence of other investigations that used the KAP survey related to skin cancer prevention, specifically in older adults. Another limitation was the fact that the prevalence of skin cancer was self-reported by the respondents, as many lesions in the early stages are not seen as skin cancer, which may favor the non-demand for health services.

### Contributions to nursing, health, and public policies

With the results of this study, it is evident the need to provide educational campaigns aimed at older adults on measures to prevent skin cancer, as this is a vulnerable public to this pathology.

It is believed that the monitoring of associated factors will be able to subsidize political and assistance changes in elder care, in the Brazilian scenario, with the aim of favoring actions that support the quality of life of this public.

## CONCLUSIONS

It is concluded that older adults’ KAP regarding skin cancer prevention were influenced by several factors, such as being between 60 and 69 years old, having incomplete elementary school, working or already working under direct exposure to the sun, having problems with pigmented spots/plaques with crusts and bleeding wounds as well as having light skin, eyes and hair.

The research identified the need for educational actions aimed at the prevention and early recognition of skin changes suggestive of neoplasm in older adults, considering the susceptibility to the disease due to the aging process. Health education on the subject can be carried out in different care settings, involving older adults and the family. In this context, new studies are essential to direct campaigns and actions, aiming at improving KAP as well as changing older adults’ behavior.
